# Supplementary material for: Design of synthetic epigenetic circuits featuring memory effects and reversible switching based on DNA methylation
Source: Nat Commun. 2017 May 24;8:15336. doi: 10.1038/ncomms15336 (PMC5458116; doi:10.1038/ncomms15336)
Supplement: Supplementary Information — Supplementary Figures, Supplementary Tables, Supplementary Note, and Supplementary References [file ncomms15336-s1.pdf]

Supplementary Figures

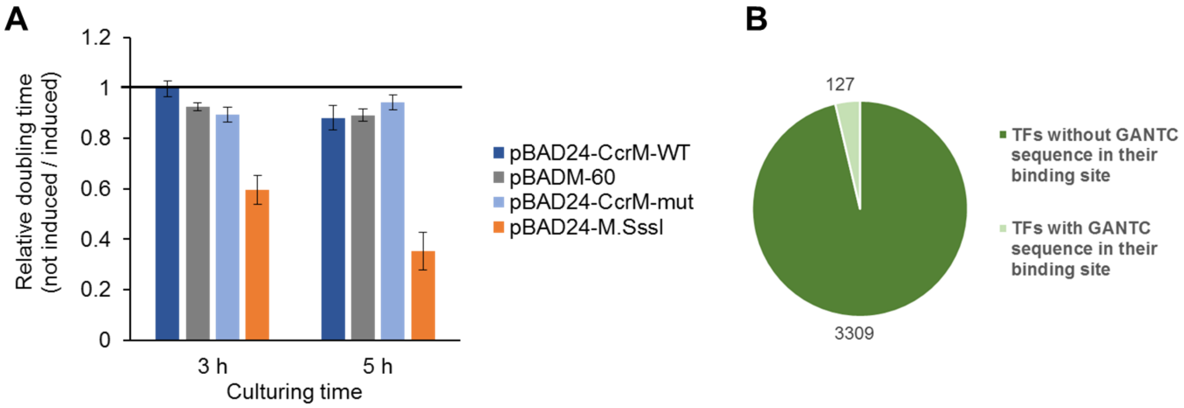

Supplementary Figure 1. Influence of CcrM expression on the viability of *E. coli*. A) Normalized doubling time of *E. coli* XL1-Blue cells transformed with pBAD vectors encoding different inducible proteins. pBAD24-CcrM-WT encodes CcrM from *Caulobacter crescentus*, pBADM-60 encodes the NusA-His tag, pBAD24-CcrM-mut encodes the catalytically inactive D31A CcrM mutant, pBAD24-M.SssI encodes the M.SssI CpG specific DNA-(cytosine C5)-methyltransferase from *Spiroplasma* sp.[1]. Cells were cultured under not inducing conditions (30°C, LB, ampicillin, 0.2% glucose) and under inducing conditions (LB, ampicillin, 0.2% arabinose). Doubling times (n=4) under inducing and non-inducing conditions were calculated from OD_{600nm} measurements, error bars indicate s.e.m.. B) Fraction of *E. coli* transcription factors[2] with CcrM site in their core recogniton sequence.

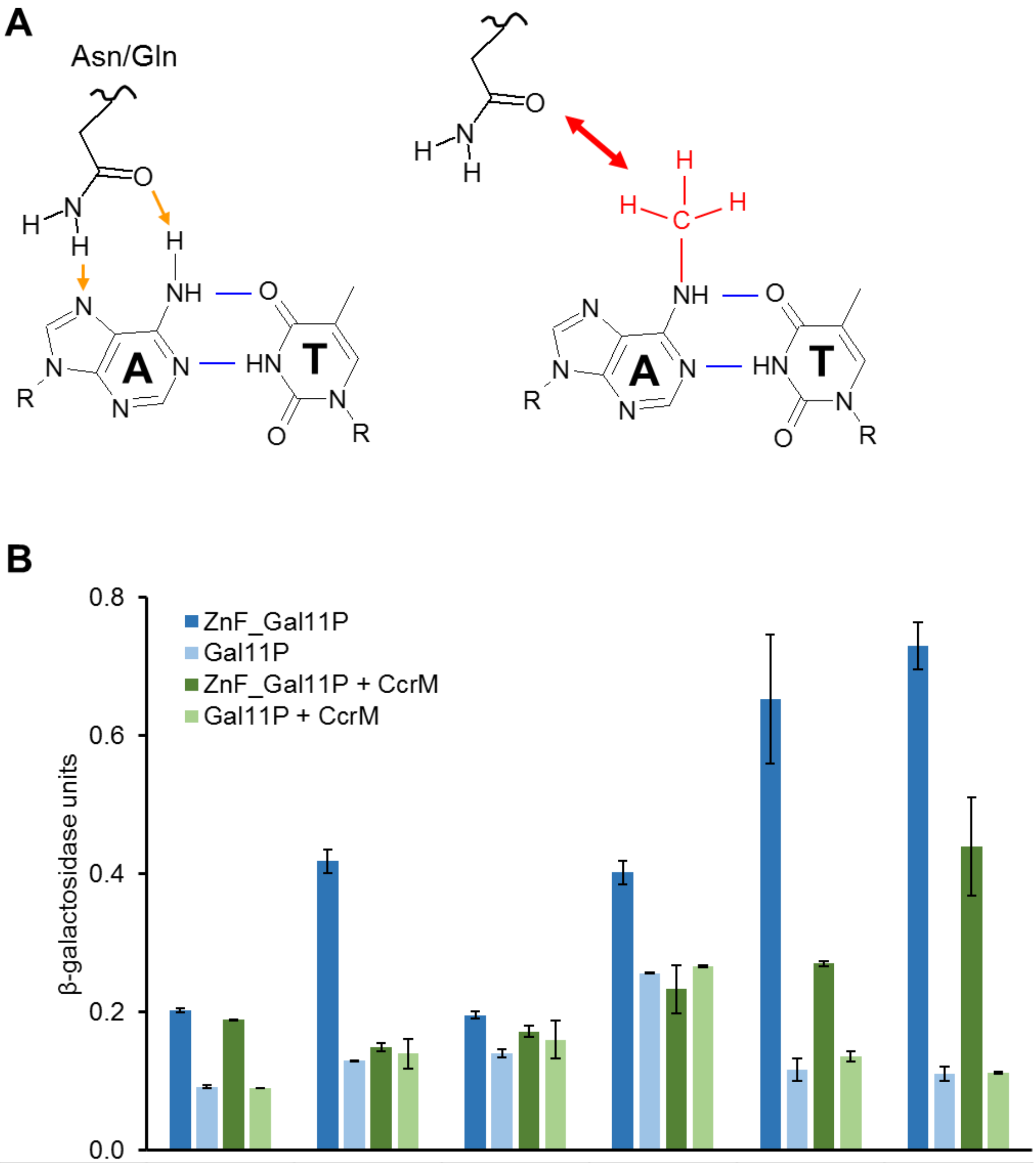

ZF system	control	ZF_1012	ZF_604	ZF_567	ZF_1026	ZF_916
ZFBS (5' to 3')	GCA GAA GCC	GGA GAA GAA TC	GAA GGA TTC	TGG GAG TCT	GGA GTA GAG TC	GAA GAC GAA TC
Finger F1	DRSSLTR	QASNLTR	RPNHLAI	SKPNLKM	KHSNLTR	QRSNLAR
Finger F2	QGGNLVR	QQTNLTR	QSAHLKR	RQDNLGR	QRSSLVR	DRGNLTR
Finger F3	QAATLQR	QTTHLSR	QSNNLTR	RMDHLAG	QTTHLSR	QSNNLTR

Supplementary Figure 2. Design of DNA-(adenine N6)-methylation specific ZnF proteins. A) Principle of the design. Asn and Gln residues can recognize unmethylated AT base pairs (left part), as predicted more than 40 years ago[3] and experimentally confirmed afterwards[4,5]. In our work, we exploit the fact, that adenine-N6 methylation disrupts this interaction (right part). B) DNA binding and methylation specificity of designed ZnF proteins analyzed with a bacterial two-hybrid system[6], in which DNA binding of the ZnF-Gal11P fusion proteins transcriptionally

activates the *lacZ* reporter gene in a quantifiable manner. Gene activation of ZnF_Gal11P fusion proteins (dark blue bars) was tested in comparison to background signals measured for Gal11p in absence of ZnF (light blue bar). The DNA binding behavior of ZnF proteins was also tested under conditions in which the DNA-(adenine N6)-methyltransferase CcrM was expressed. The dark green bars show the ß-galactosidase activity in the presence of ZnF_Gal11p and CcrM, and the light green bars show the background ß-galactosidase activity in the presence of CcrM. The error bars indicate the s.d. of two biological replicates.

As a control, a ZnFinger_Gal11p fusion protein and its corresponding binding site was used, which does not include a CcrM recognition site. The control was provided as part of the used Addgene Kit #1000000010 to test ZnF protein binding. The tested ZnF binding sites (ZFBS) are underlined, CcrM target sequences are overlined and methylatable adenines are shown in bold. The amino acid sequences of the ZnF protein's alpha helixes forming the sequence specific DNA contacts are indicated. The Asn and Gln residues designed to be involved in binding the unmethylated adenine residue are marked in blue.

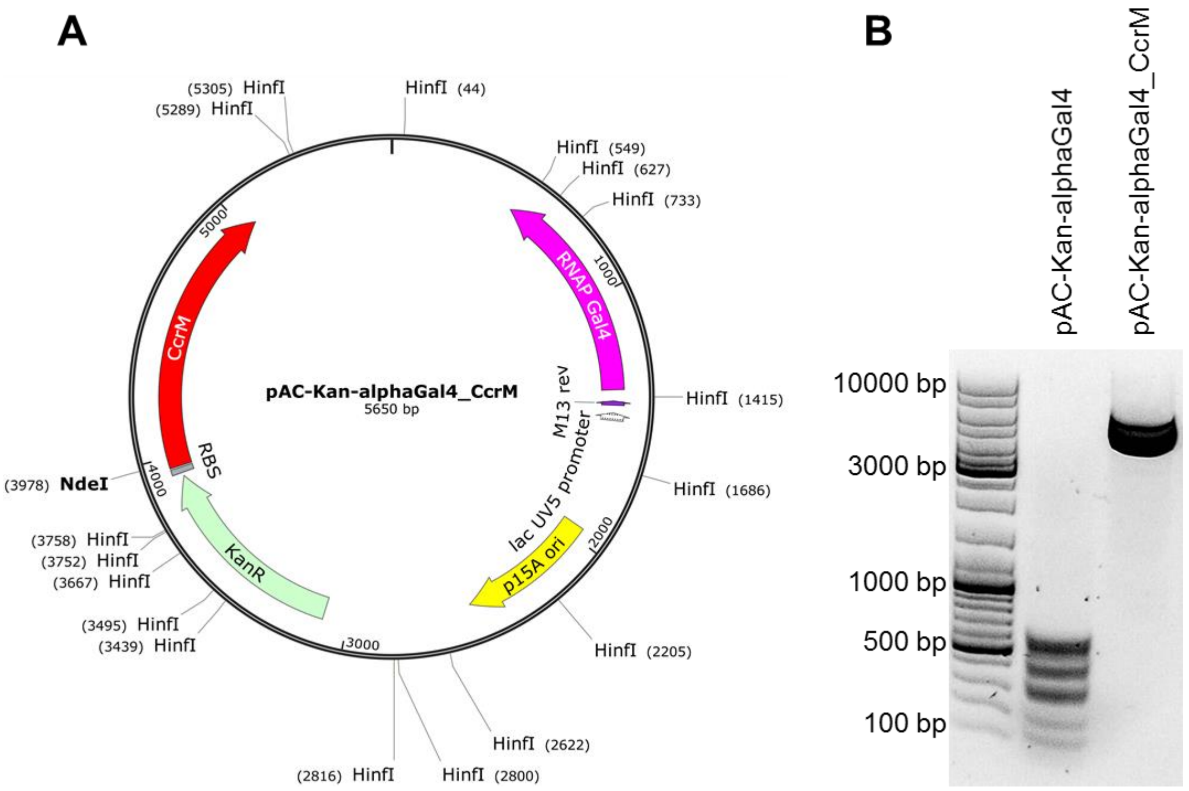

Supplementary Figure 3. Insertion of the CcrM DNA methyltransferase into the pAC-Kan-alphaGal4 plasmid of the bacterial two-hybrid system. A) Plasmid map of pAC-Kan-alphaGal4_CcrM with indicated restriction sites. B) Restriction digest of pAC-Kan-alphaGal4 and pAC-Kan-alphaGal4_CcrM plasmid DNA with the endonucleases HinfI and NdeI. HinfI only cleaves GANTC sites in an unmethylated state. The results show that the introduced CcrM gene is expressed and active in bacterial cells, because GANTC sites in pAC-Kan-alphaGal4_CcrM are protected from HinfI cleavage.

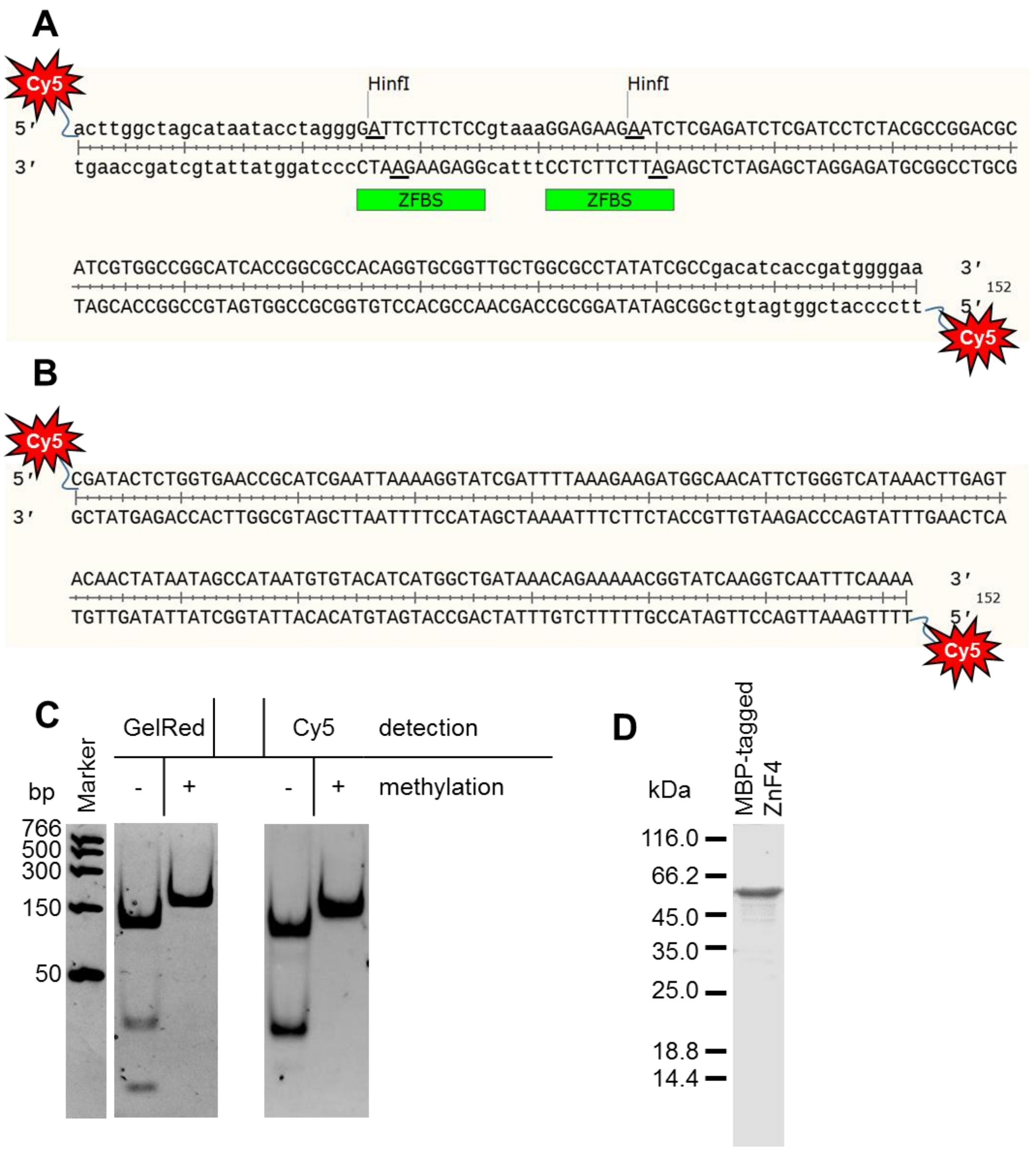

Supplementary Figure 4. EMSA substrates and quality control. A) EMSA DNA substrate sequence with zinc finger binding sites (ZFBS). Indicated are HinfI restriction sites, which are protected from cleavage by CcrM methylation of the underlined adenine base. EMSA substrates were generated by PCR using 5'-Cy5-labelled oligonucleotides as primers. This substrate was used in gel shift experiments (Supplementary Fig. 5) in unmethylated form (BS, "for binding site") and methylated form (BS-M, for "binding site methylated"). Methylation of the substrate was performed *in vitro* using purified CcrM enzyme[7,8]. B) EMSA DNA substrate sequence without ZFBS (c, for "control") in gel shift experiments (Supplementary Fig. 5). C) Digestion of EMSA substrates containing ZFBSs. *In vitro* CcrM-methylated and unmethylated DNA substrates were digested with HinfI and the products separated by 8% non-denaturing polyacrylamide gelelectrophoresis in 0.5 x TB buffer. DNA fragments were visualized by GelRed nucleic acid stain and by fluorescence detection of the Cy5 dye. Expected product sizes visible with GelRed staining are 22 bp, 25 bp, and 105 bp for unmethylated substrate, and 156 bp for the methylated substrate. Expected product sizes visible by fluorescence detection are 25

bp and 105 bp for the unmethylated substrate, and 156 bp for the methylated substrate. D) Coomassie stained SDS-PAGE of the purified MBP-tagged ZnF4 (expected size: 59.4 kDa).

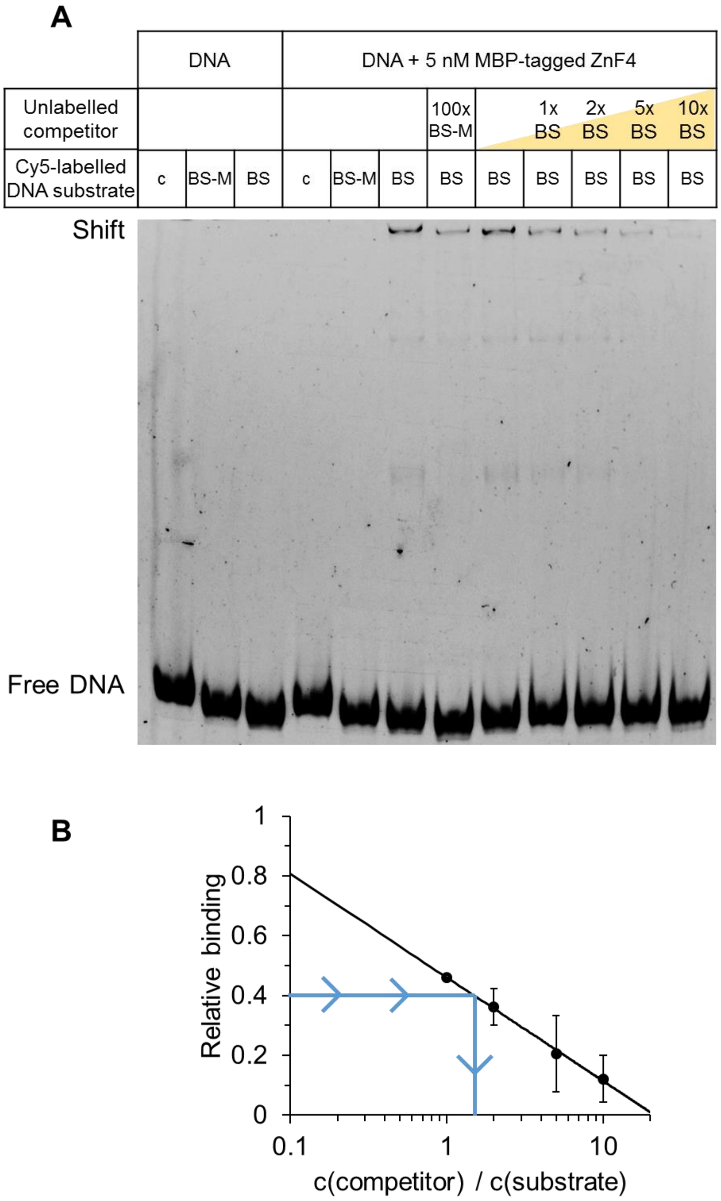

Supplementary Figure 5. A) Example of EMSA experiments studying the binding of purified MBP-tagged ZnF4 to different DNA substrates (Supplementary Fig. 4). Cy5-labelled PCR products containing no zinc finger binding site (c), a methylated zinc finger binding site (BS-M) or an unmethylated zinc finger binding site (BS) were used as binding substrates in a concentration of 5 nM. Unlabelled BS and BS-M were used as binding competitor for labelled BS. B) Relative binding of unmethylated, labelled substrate DNA in the presence of different amounts of unmethylated, unlabelled competitor DNA (BS). Relative binding refers to normalized band intensities of the gel shift. Binding competition with methylated unlabelled DNA (BS-M) revealed 59% competition at an 100x excess of BS-M, which corresponds to a 1.5x excess of unmethylated competitor (shown by the blue arrows) indicating a 67.7 (±7.3) fold preference of ZnF4 for binding to unmethylated DNA. Error bars indicate s.d., n=2.

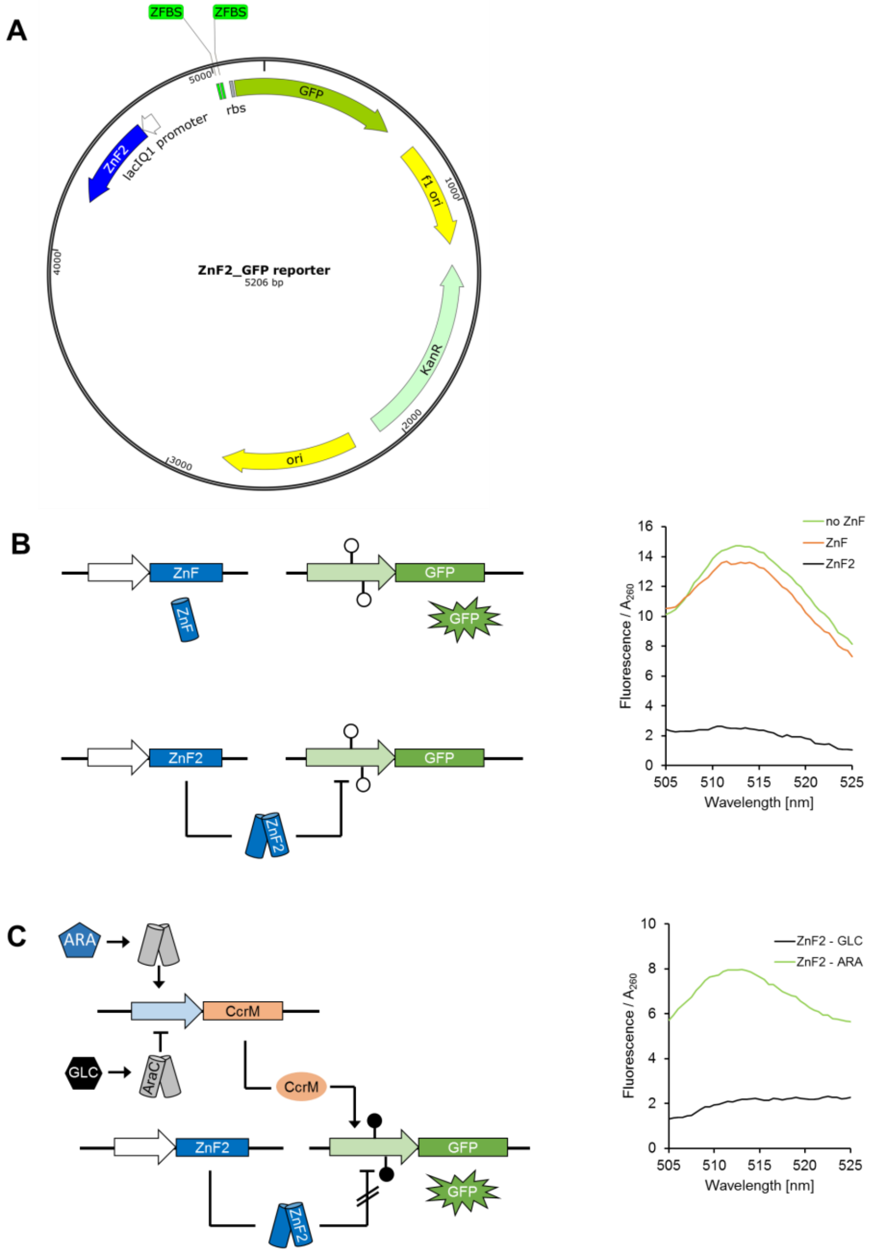

Supplementary Figure 6. Schematic representation and validation of the artificial methylation sensitive gene expression system. A) Plasmid map of the ZnF2 GFP reporter plasmid. B) Circuit designs of the artificial methylation sensitive gene expression system. The artificial ZnF repressor is constitutively expressed. The monomeric ZnF protein does not repress GFP gene expression, hence, a fluorescence signal is detected (orange trace). A dimerized version of the zinc finger protein (ZnF2), however, is able to repress GFP expression (black trace). The green trace shows GFP expression in the absence of ZnF proteins. C) Induction of the artificial methylation sensitive gene expression system. Upon arabinose supplementation CcrM is expressed and it methylates the binding sites for the ZnF repressor, weakening ZnF2 binding and leading to GFP expression (green trace). GFP expression is not detectable under repressive glucose conditions (black trace).

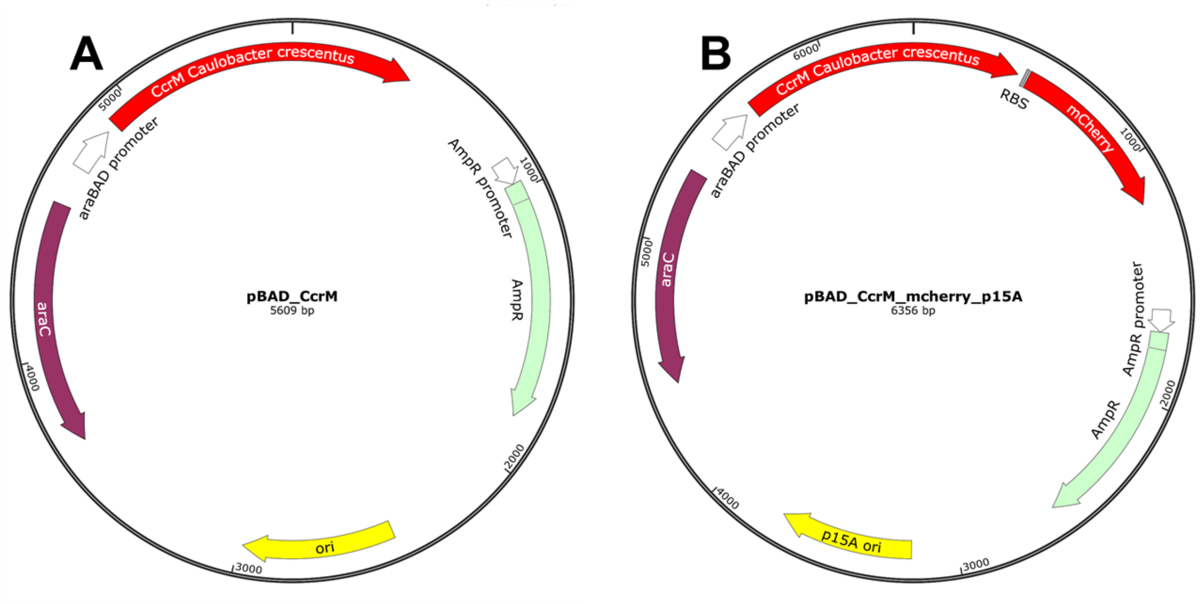

Supplementary Figure 7. Plasmid maps of the trigger plasmids for arabinose induction of CcrM used in memory system II. A) First generation of the trigger plasmid based on pBAD24 plasmid containing *ccrM* gene. B) Second generation of the arabinose trigger plasmid. The pBAD24 plasmid containing a *ccrM* gene was modified by replacing the pBR322 origin of replication with a p15A origin of replication in order to propagate the plasmid in *E. coli* cells containing a second plasmid with a pBR322 origin of replication. The gene for the red fluorescent protein mCherry was introduced downstream of the *ccrM* gene with its own ribosome binding site generating a polycistronic operon.

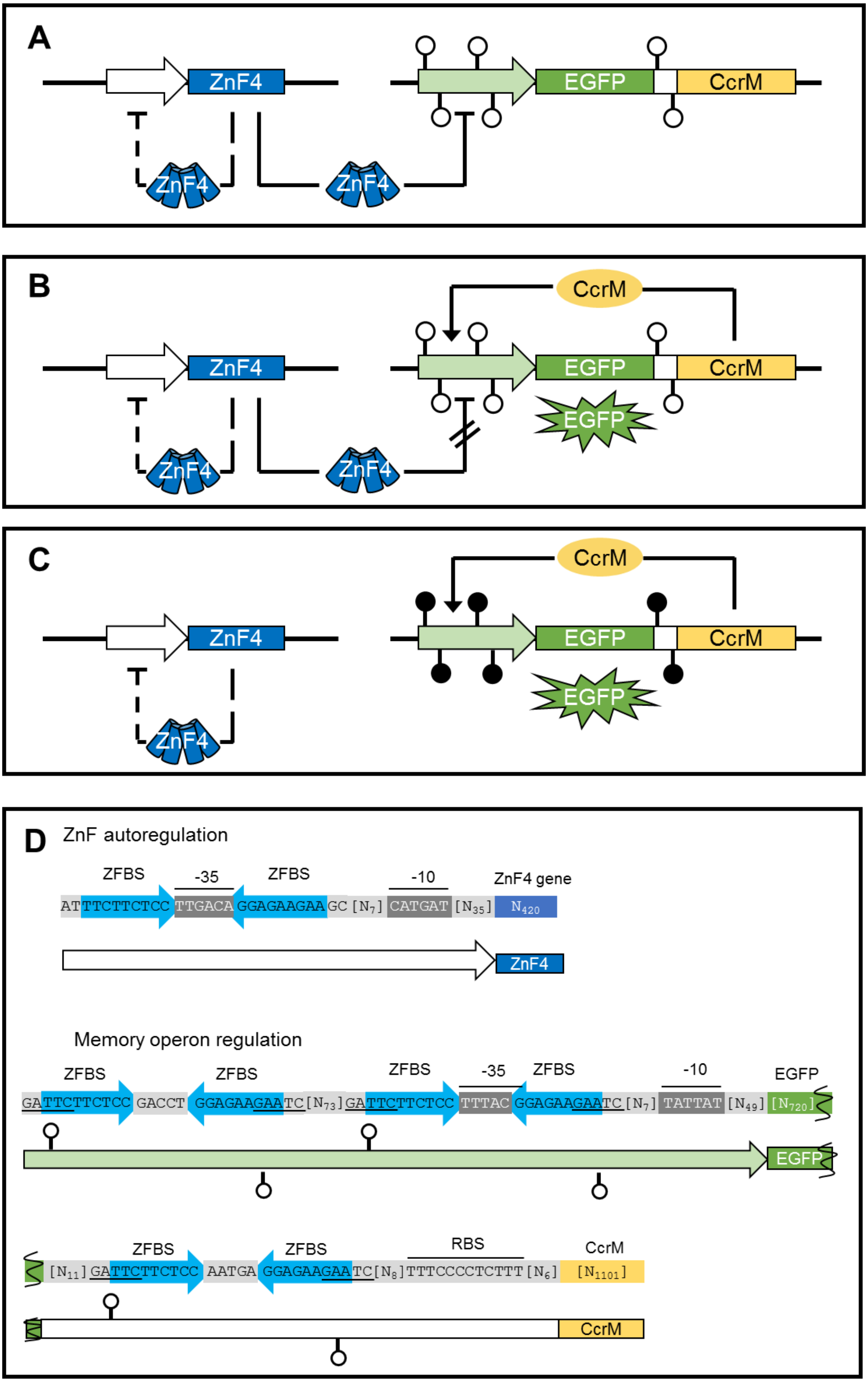

A
ZnF4
ZnF4
ZnF4
EGFP
CcrM
B
CcrM
ZnF4
EGFP
CcrM
EGFP
ZnF4
ZnF4
C
CcrM
ZnF4
EGFP
CcrM
EGFP
ZnF4
D
ZnF autoregulation
ZFBS
-35
ZFBS
-10
ZnF4 gene
ATTTCTTCTCC TTGACA GGAGAAGAAGC [N$_{7}$] CATGAT [N$_{35}$] N$_{420}$
ZnF4
Memory operon regulation
ZFBS
ZFBS
ZFBS
-35
ZFBS
-10
EGFP
GATTCTTCTCC GACCT GGAGAAGAATC [N$_{73}$] GATTCTTCTCC TTTAC GGAGAAGAATC [N$_{7}$] TATTAT [N$_{49}$] [N$_{720}$]
EGFP
ZFBS
ZFBS
RBS
CcrM
[N$_{11}$] GATTCTTCTCC AATGA GGAGAAGAATC [N$_{8}$] TTTCCCCTCTTT [N$_{6}$] [N$_{1101}$]
CcrM

Supplementary Figure 8. Physical induction of the memory system I by heat. A) Scheme of the memory system in the off-state during cultivation at 30°C. The ZnF4 repressor binds the promoter region of the reporter-maintenance operon and represses transcription because the ZnF binding sites are in an unmethylated state (empty lollipops). The ZnF4 repressor negatively regulates the transcription of its own gene (dashed lines). This autoinhibition is unaffected by CcrM expression, because the ZnF binding sites in the ZnF4 promoter do not overlap with a GANTC site. B) Switching into the on-state. Upon a temperature shift to 37°C, binding of the ZnF4 repressor to its binding sites is weakened, which leads to expression of the reporter-maintenance operon. C) Stable on-state propagation. The CcrM methyltransferase methylates the ZnF binding sites and hinders DNA binding of the ZnF4 protein. DNA methylation lost by DNA replication is immediately restored by CcrM, resulting in a positive feedback loop (memory function). D) Annotated DNA sequences of the promoter regions of the ZnF protein and the memory operon. Zinc finger binding sites are indicated with blue arrows, -35 and -10 regions as dark grey boxes and the genes for ZnF4, EGFP and CcrM are represented as colored boxes, similarily as drawn in panels A-C.

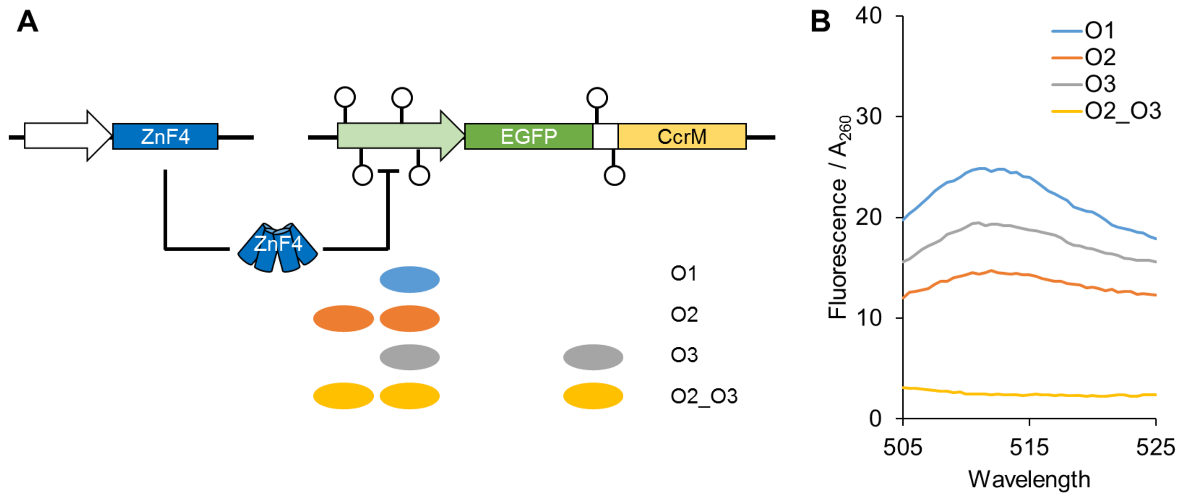

Supplementary Figure 9. Efficiency of different ZnF binding site arrangements for off-state maintenance. A) Scheme of the memory system with indicated palindromic double binding sites. O1 contains one double binding site, similarly to the methylation sensitive gene expression system in Supplementary Fig. 6. The O2 construct has an additional double binding site 100 bps upstream. O3 harbors the double binding site O1 and additionally one double binding site in the intergenic region just upstream of the CcrM gene. The O2_O3 construct includes all three binding sites. B) Fluorescence measurements of 30°C overnight cultures containing the different memory plasmids. All three palindromic double binding sites are necessary for stable and efficient repression of the memory system allowing to maintain an off-state with no detectable fluorescence.

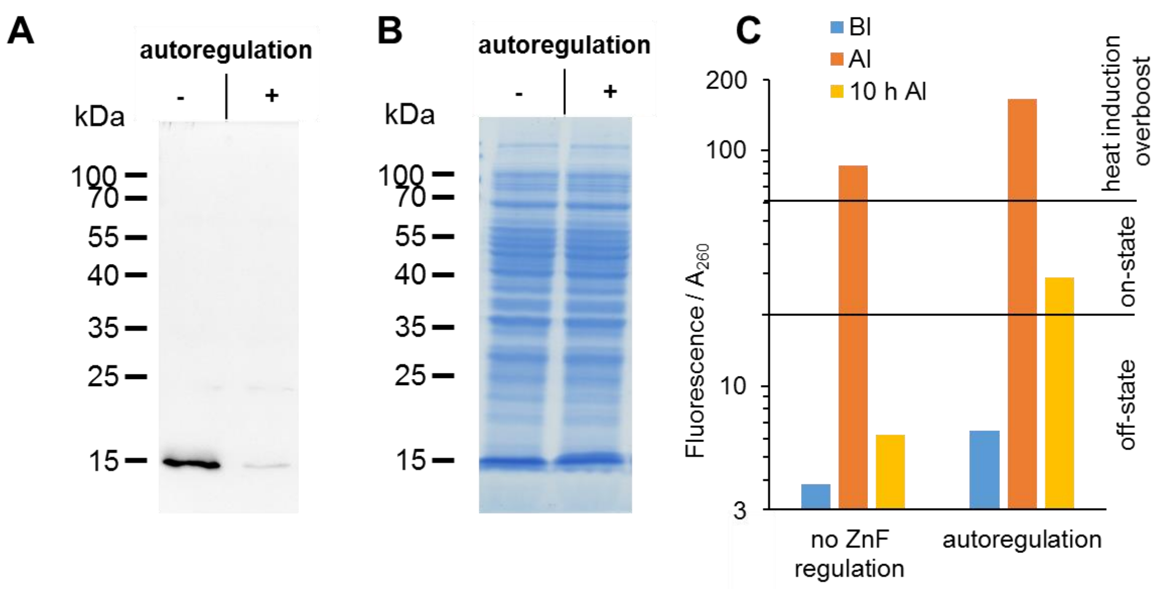

Supplementary Figure 10. Autoregulation of the ZnF repressor protein expression. In order to achieve a regulated expression of the ZnF repressor protein (ZnF4), two binding sites for ZnF_1012 not overlapping with GANTC sites were introduced into the promoter region of ZnF4. A) We studied the effect of the autoregulation of ZnF4 by comparison of the protein expression level of FLAG tagged ZnF4 with (lanes labelled with +) and without autoregulation (lanes labelled with -) by Western Blot. XL1-Blue cells transformed with the memory-maintenance plasmid with and without autoregulation of ZnF4 were grown overnight at 30°C in LB medium supplemented with kanamycin and cells were harvested by centrifugation. Protein extracts were prepared by suspending 0.5 OD_{600nm} cells in 2 x SDS-PAGE loading buffer followed by incubation at 95°C for 10 minutes. Total cell lysates were separated by polyacrylamide gel electrophoresis on a 15% SDS-Polyacrylamide gel. Proteins were transferred onto a nitrocellulose membrane and the membrane was blocked by incubation in PBST containing 5% skim milk for 2 h at room temperature. After washing, the blot was incubated with primary anti FLAG antibody (Sigma, SLBN5629V) for 1 h and washed again before incubating with secondary anti mouse antibody coupled to horseradish peroxidase (GE Healthcare, 370149). Detection was performed with enhanced chemiluminescent substrate (Pierce® ECL Western Blotting Substrate) following the manufacturer's recommendations and a FUSION Solo (VWR International) system was used for signal recording. The western blot of the synthetic FLAG tagged ZnF4 repressor (theoretical size: 16.6 kDa) shows a much lower expression of ZnF4 under autoregulation conditions. B) Coomassie stained SDS-PAGE serving as a loading control for panel A. C) Fluorescence measurements of memory system I with and without autoregulation of the ZnF4 repressor (BI, before induction; AI, after induction; 10 h AI, 10 h after induction). 10 h after heat induction of the memory system, the system returned to the off-state without autoregulation of the ZnF repressor, presumably because of high concentrations of the repressor protein blocking access of CcrM. In contrast, a stable on-state was observed with autoregulation of the ZnF repressor.

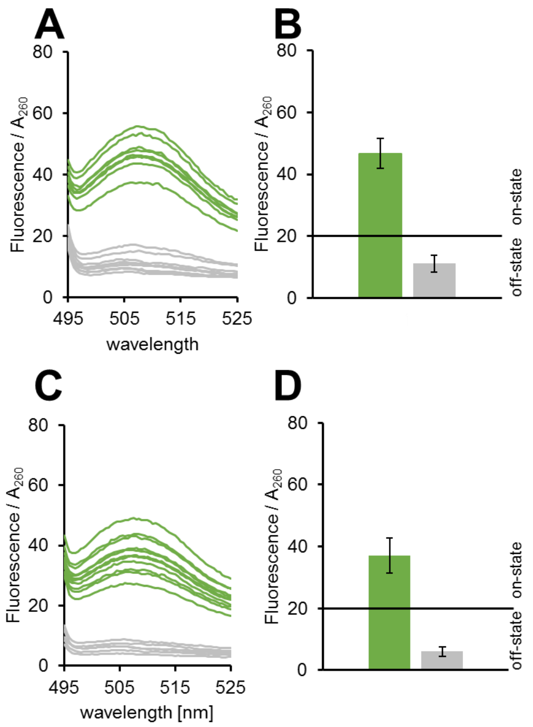

Supplementary Figure 11. Stability of the on-state of memory systems I and II. A) XL1-Blue cells with the memory system I were switched to the on-state by heat induction. After 48 h cultivation in liquid culture at 30°C, cells were plated to single colonies on LB agar plates containing appropriate antibiotics. After incubation for additional 48 h on agar plates at 30°C, individual colonies were used to inoculate overnight cultures and EGFP fluorescence intensity was measured in cell extracts after OD_{260nm} normalization (green traces). The results indicate that the on-state was maintained in all analyzed colonies. Grey traces are not induced overnight cultures propagated in the off-state. B) Average fluorescence signal of the experiment shown in panel A (green bar: on-state cultures, n=10, error bars indicate s.d.) and off-state for comparison (grey bar: off-state cultures, n=9, error bars indicate s.d.). C) XL1-Blue cells with memory system II were induced with arabinose and 24 h AI competent cells were prepared from this culture. Cells were mock transformed with 5 ng of PCR product and plated on LB agar plates containing kanamycin, ampicillin, and glucose at 30°C. Overnight cultures were inoculated from individual colonies and EGFP expression analysed as described in A). All analysed cultures had maintained the on-state (green traces). Grey traces represent fluorescent signals recorded with off-state cultures containing memory system II. D) Average fluorescence signal of the experiment shown in panel C (green bar: on-state cultures, n=12, error bars indicate s.d.) and off-state negative control (grey bar: off-state cultures, n=6, error bars indicate s.d.).

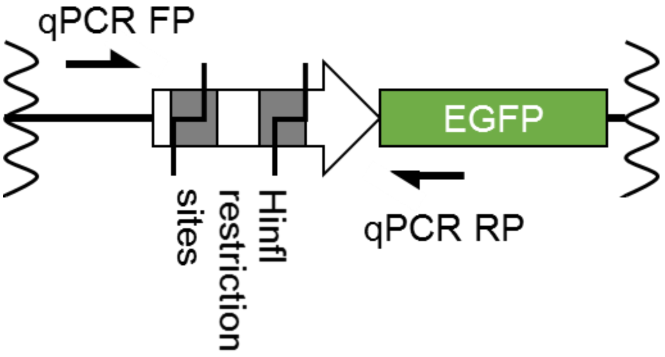

Supplementary Figure 12. Principle of the methylation analysis of the reporter-maintenance operon promoter. Schematic view of the plasmid region analyzed by quantitative PCR with indicated primers. Prior to qPCR, the plasmid DNA was digested with HinfI, which cleaves DNA at unmethylated GANTC sites. “Methylation” refers to DNA molecules which are not cleaved by HinfI. As an internal standard, a reference amplicon in the kanamycin resistance gene without HinfI restriction site was used.

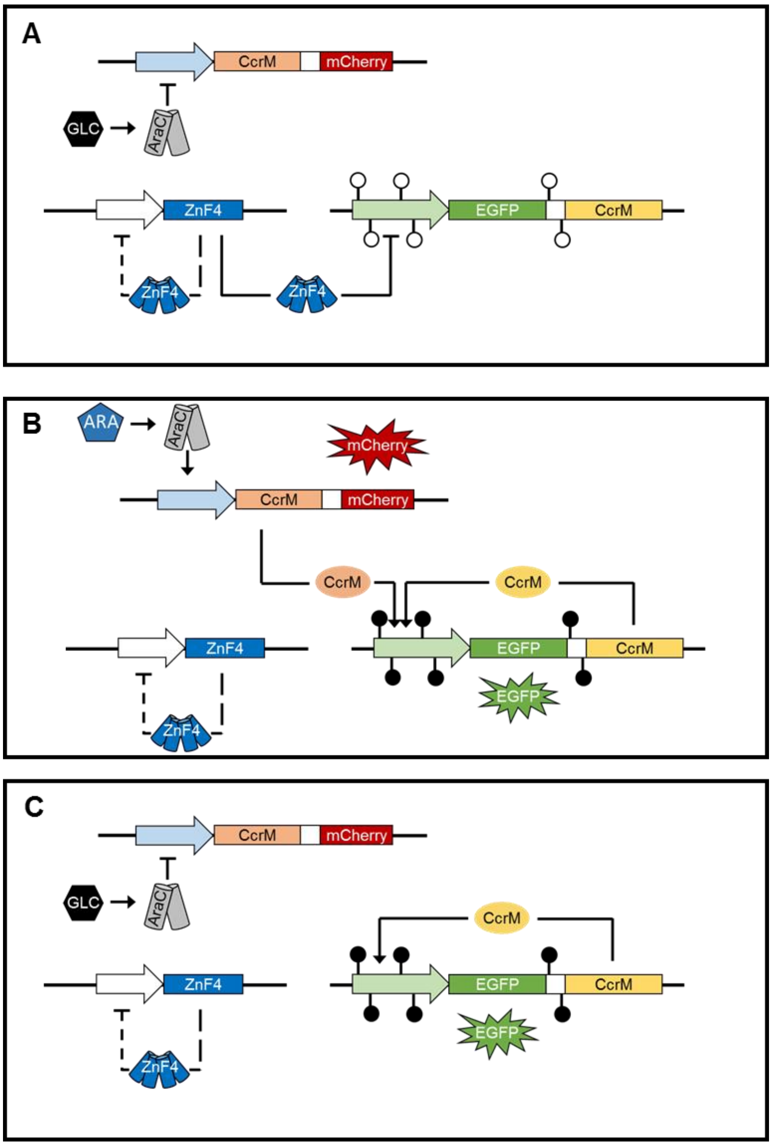

Supplementary Figure 13. Chemical induction of the memory system II by arabinose addition. A) In the off-state, AraC represses the trigger operon including the trigger MTase (orange CcrM) and mCherry reporter. The reporter-maintenance operon encoded on the memory plasmid is repressed by the ZnF4 repressor, which binds to its unmethylated binding sites (empty lollipops). B) Induction of the on-state by arabinose induction. AraC activates the P_{BAD} promoter, which subsequently leads to the expression of the trigger MTase and mCherry. The trigger MTase methylates the ZnF binding sites on the reporter-maintenance operon (filled lollipops) and hinders ZnF4 binding. The EGFP reporter and the maintenance MTase (yellow CcrM) are expressed. C) Stable perpetuation of the on-state. After switching the medium to glucose containing medium, the trigger operon is repressed again. However, the reporter/maintenance operon is still being expressed due to constant remethylation of the operator sites by the maintenance MTase.

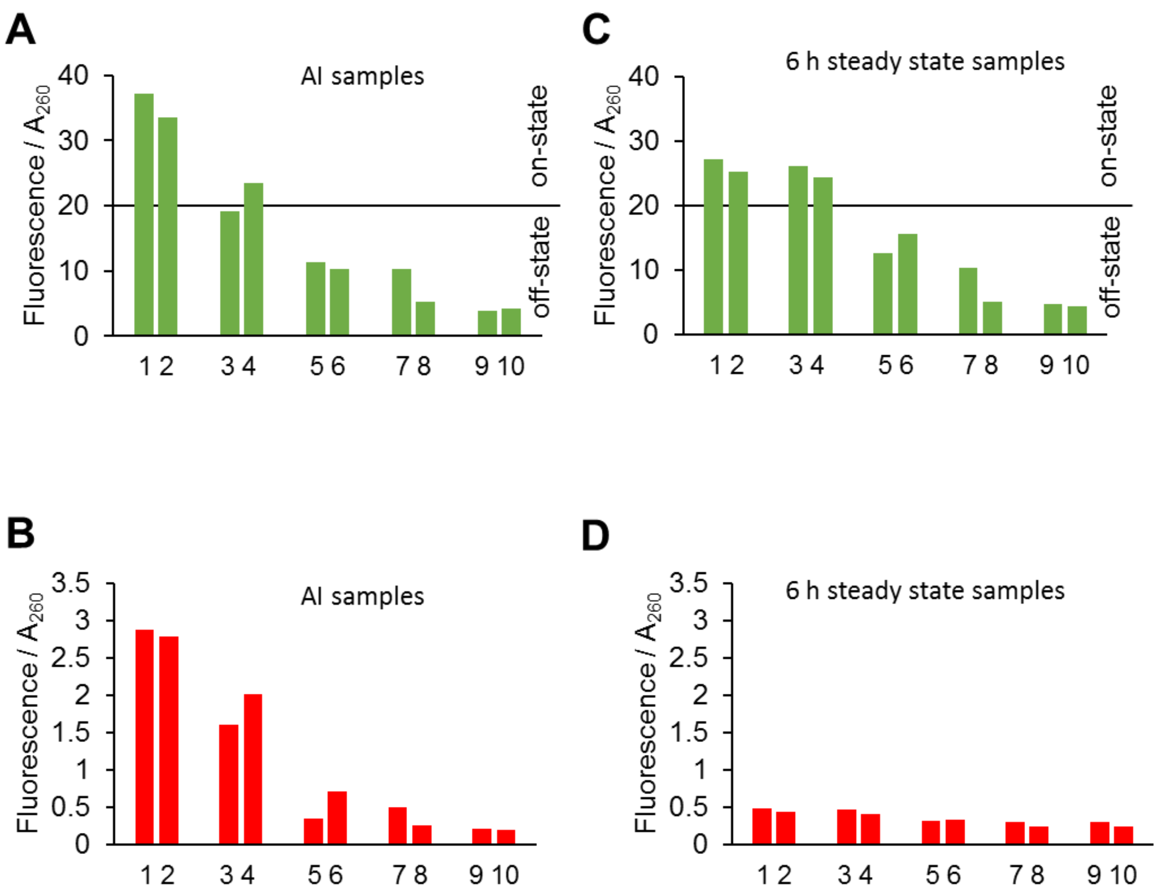

Supplementary Figure 14. Characterization of memory system II response at different arabinose concentrations in the culturing medium after overnight induction. A) EGFP fluorescence measurements of cells, which were cultured overnight in media containing 0.2% (samples 1 and 2), 0.01% (samples 3 and 4), or 0.0005% arabinose (samples 5 and 6), no additional sugar (samples 7 and 8), or 0.2% glucose (samples 9 and 10). B) mCherry measurements of the same samples from panel A. C) Bacterial cultures analyzed in panels A and B were transferred to medium containing 0.2% glucose but no arabinose. Cells were cultured for 6 h and fluorescent measurements for EGFP fluorescence were performed. D) mCherry fluorescence measurements of the same samples as in panel C. The data show that cells cultivated in ≥ 0.01% arabinose switched to the stable on-state. The low mCherry signal indicates that the on-state was maintained by the memory system.

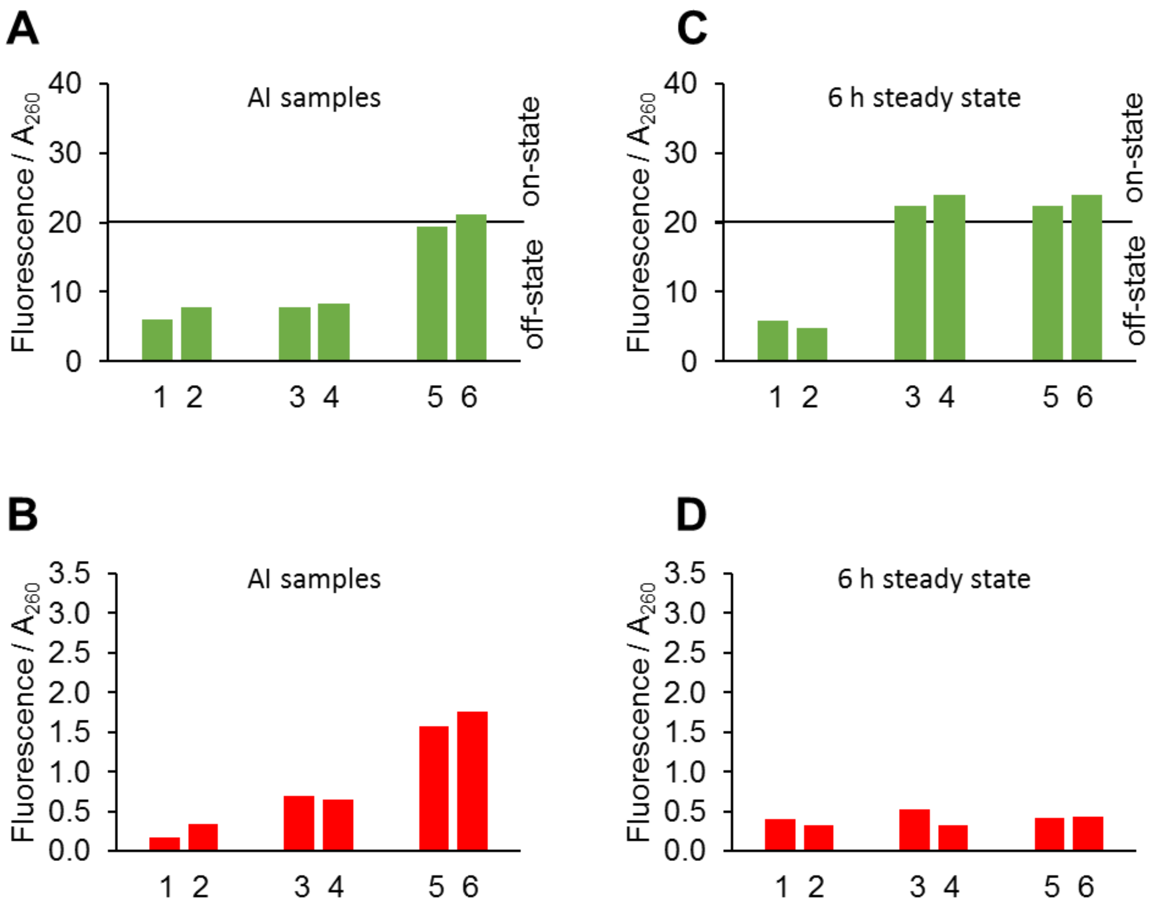

Supplementary Figure 15. Influence of different induction durations on memory system II on-switching. Experiments were conducted with 0.2% arabinose in the medium. A) EGFP fluorescence measurements of cells harboring memory system II which have been cultured with 0.2% glucose (samples 1 and 2). Samples 3-6 were cultivated in medium containing 0.2% arabinose for 2 h (samples 3 and 4) or 4 h (samples 5 and 6). B) mCherry measurements of the same samples as in panel A. C) EGFP measurements of the samples from panel A after cultivation for 6 h in 0.2% glucose. The data reveal switching to the on-state for samples 3-6. D) mCherry measurements of the same samples from panel C. The data show that cells induced for more than 2 h with 0.2% arabinose switched to the stable on-state. The low mCherry signal indicates that the on-state was maintained by the memory system.

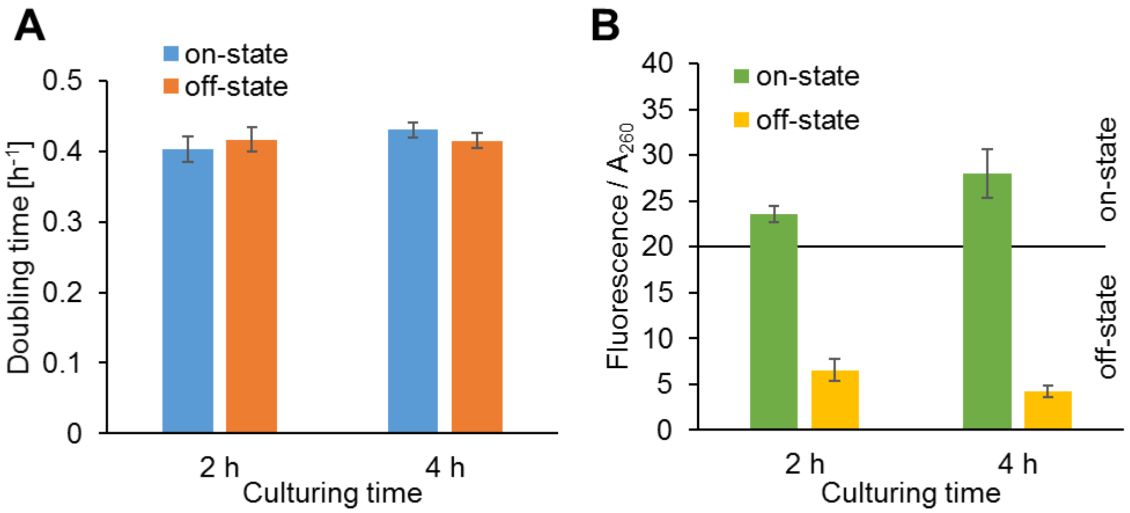

Supplementary Figure 16. Growth rates and fluorescence signal of XL1-Blue cells harboring memory system II in the on-state and the off-state. A) Growth rates do not differ between cells in the on-state and cells in the off state and at different culturing times. Cells were cultured in medium under not inducing conditions (0.2% glucose) with appropriate antibiotics in exponential growth phase. Doubling times were calculated from OD_{600nm} measurements. B) EGFP fluorescence signals of the cultures used to determine the growth rates which are shown to confirm their on- and off-state (error bars indicate s.d., n=2).

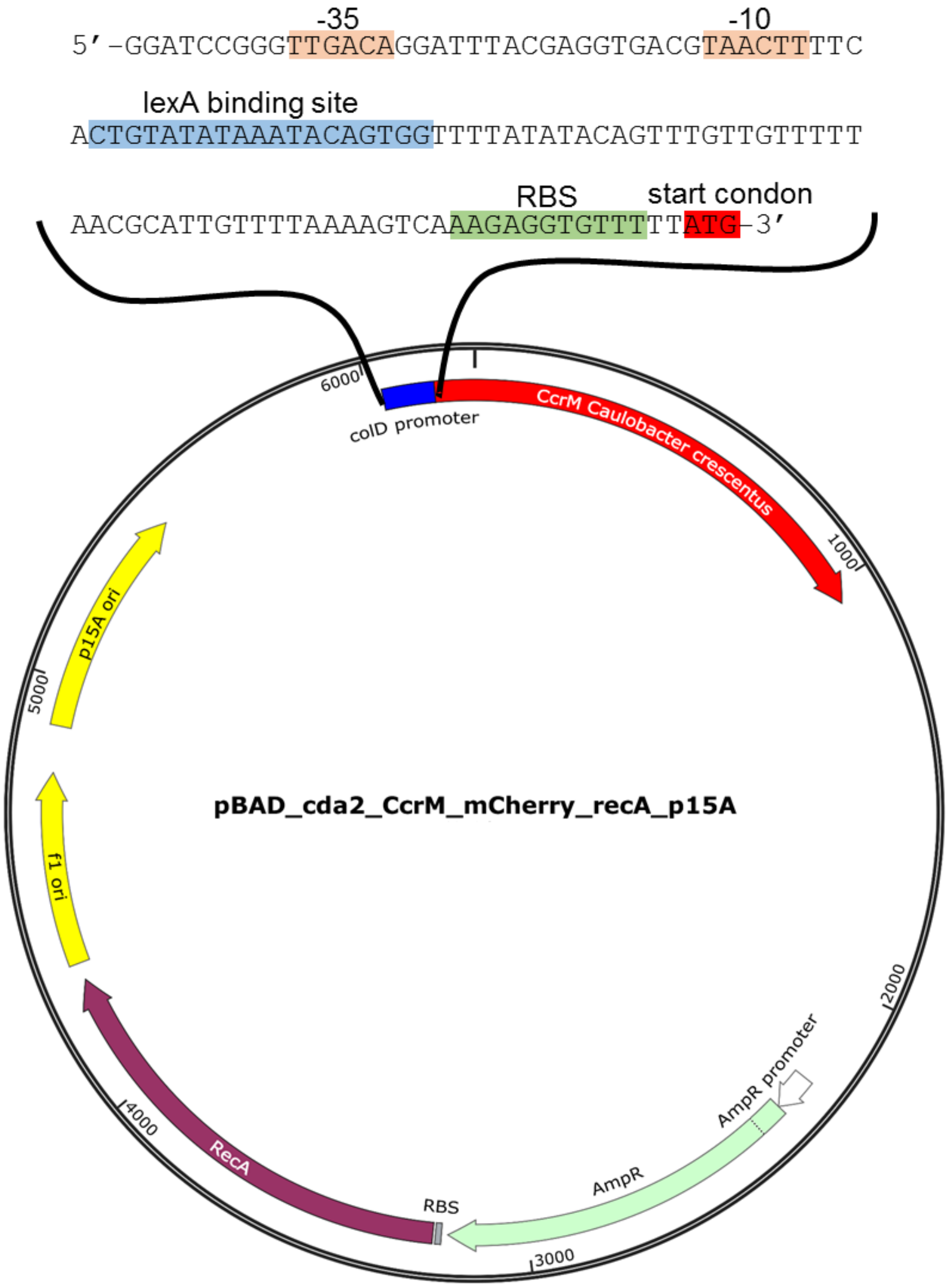

Supplementary Figure 17. Plasmid map of the DNA damage sensor plasmid used in memory system III. The plasmid is derived from the pBAD24 vector carrying the *Caulobacter crescentus* CcrM gene. The pBR322 origin of replication of the pBAD24 vector was replaced by the p15A origin of replication. The pBAD promoter and the araC gene were removed and replaced by the colD promoter, which is modified from the promoter region of the *cda* gene in the *E. coli* plasmid pColD-157 (Genbank: Y10412.1). As *E. coli* XL1-Blue cells are recA^{-}, a copy of wild-type recA was introduced downstream of the AmpR gene with its own ribosome binding site creating a polycistronic operon.

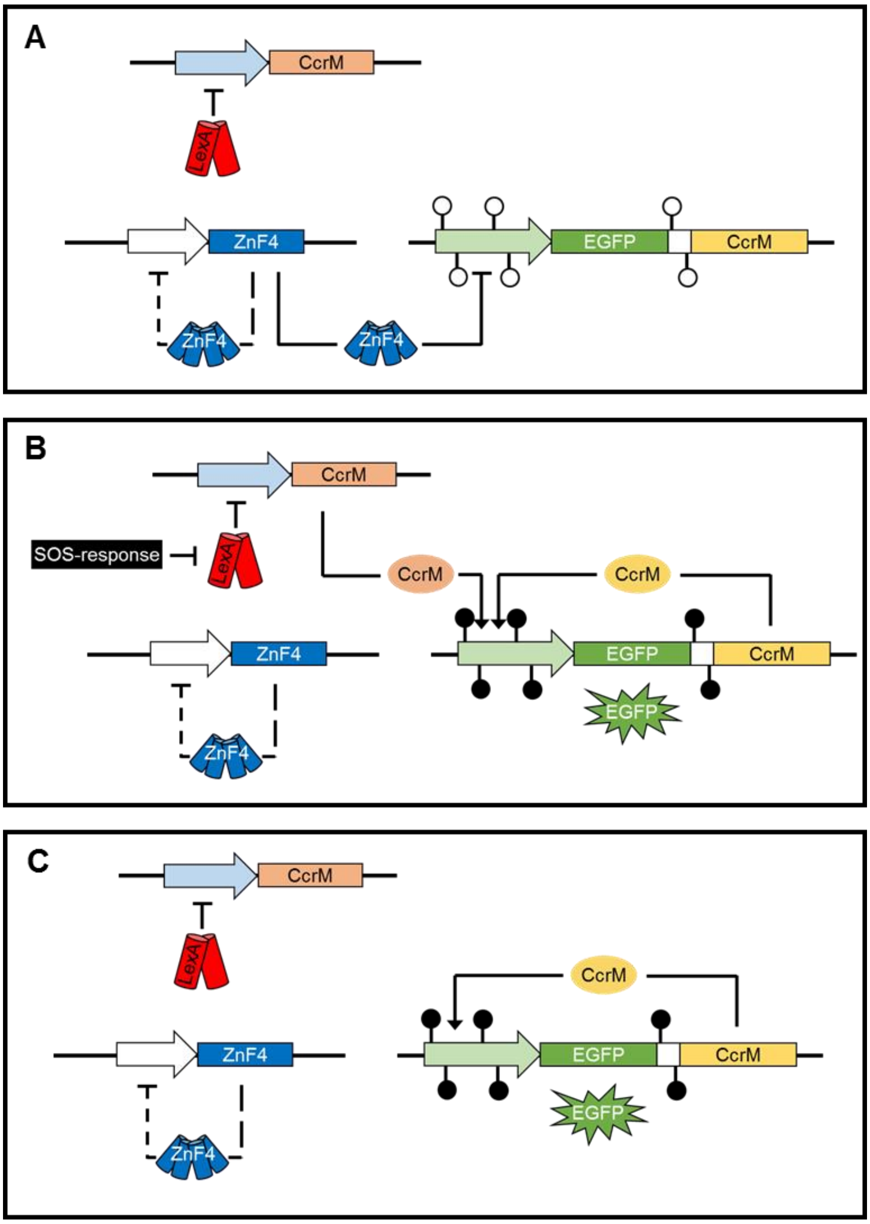

Supplementary Figure 18. Possible states of the DNA damage memory system III. A) LexA represses transcription of the trigger MTase (orange CcrM) in the off-state. Unmethylated ZnF binding sites (empty lollipops) in the memory plasmid are bound by the ZnF4 repressor. B) Switch to the on-state by SOS-response. Triggered by RecA, LexA undergoes self-cleavage and no longer represses the transcription of the trigger MTase. The trigger MTase methylates the ZnF binding sites on the memory plasmid (filled lollipops), which hinders ZnF4 binding and allows transcription of EGFP and the maintenance MTase (yellow CcrM). C) Stable propagation of the on-state after termination of SOS-response. Although LexA represses transcription of the trigger MTase, the maintenance MTase keeps the ZnF binding sites on the memory plasmid in a methylated state and the system stays in the on-state.

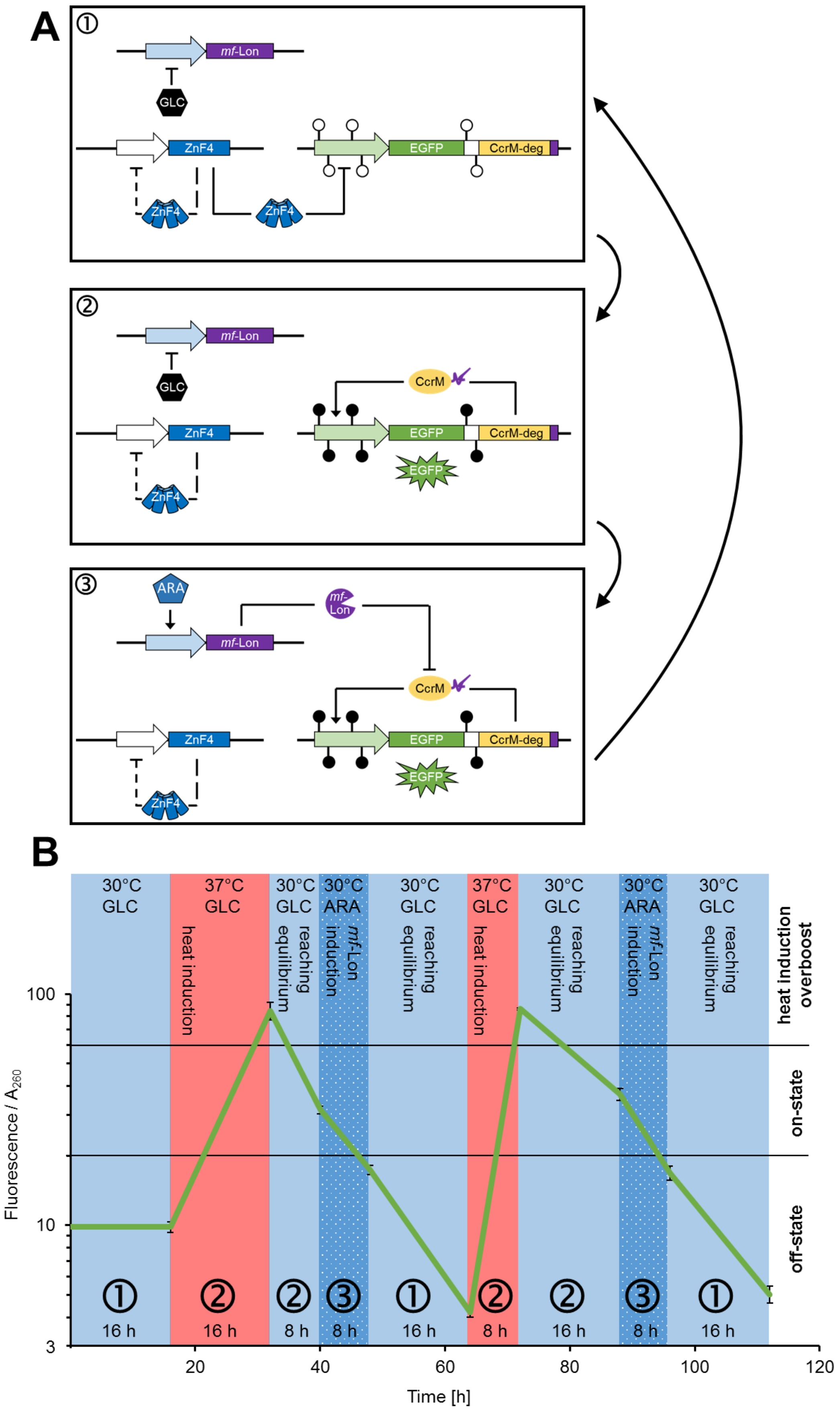

A
mf-Lon
GLC
ZnF4
EGFP
CcrM-deg
CcrM
ARA
mf-Lon
B
30°C GLC
37°C GLC
30°C GLC
30°C ARA
heat induction
reaching equilibrium
mf-Lon induction
heat induction overboost
on-state
off-state
16 h
8 h
Fluorescence / A_{260}
Time [h]
100
10
3
20
40
60
80
100
120

Supplementary Figure 19. Reset of memory system IV by protein degradation. A) Schematic drawing of the states of the reversible memory system. ①: Initial or induced off-state. ②: The memory system is switched in the on-state by overnight heat-induction (37°C). After transferring the cells back to 30°C and letting them reach a stable on-state, *mf*-Lon is induced by arabinose supplementation. ③: Subsequently, the off-state is reached, leading to a drop in EGFP fluorescence levels. B) EGFP fluorescence data of switching cycles of the reversible memory system using the maintenance CcrM MTase carrying a specific *mf*-Lon degradation tag. The memory system was induced by heat. The *mf*-Lon protease (induced by arabinose) was used to degrade tagged CcrM (CcrM-deg) and reset the system. The off-state was also maintained after switching back to glucose containing media, no longer expressing *mf*-Lon. It was possible to switch the system to the on-state again and subsequently switch it off again by *mf*-Lon expression in one continuous culture (error bars indicate s.d., n=3). For controls see Supplementary Fig. 20.

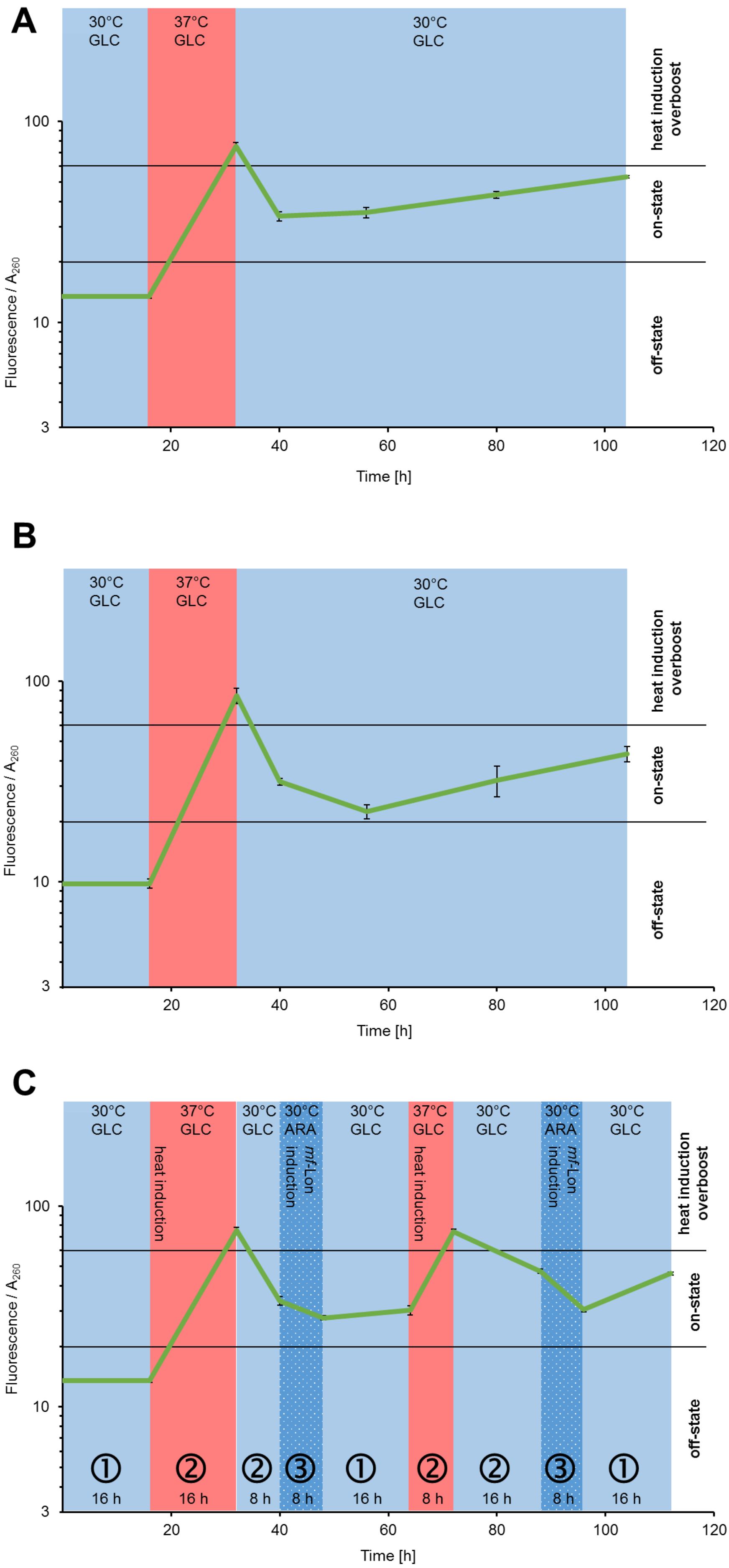
A
30°C GLC
37°C GLC
30°C GLC
heat induction overboost
on-state
off-state
Fluorescence / A_{260}
Time [h]
B
30°C GLC
37°C GLC
30°C GLC
heat induction overboost
on-state
off-state
Fluorescence / A_{260}
Time [h]
C
30°C GLC
37°C GLC
30°C GLC
30°C ARA
30°C GLC
37°C GLC
30°C GLC
30°C ARA
30°C GLC
heat induction
mf-Lon induction
heat induction
mf-Lon induction
heat induction overboost
on-state
off-state
① 16 h
② 16 h
② 8 h
③ 8 h
① 16 h
② 8 h
② 16 h
③ 8 h
① 16 h
Fluorescence / A_{260}
Time [h]

Supplementary Figure 20. Signal reset control experiments. A) Switched on memory system with untagged CcrM. The system remains in the on-state during the entire observation time. B) Switched on memory system with tagged CcrM but without *mf*-Lon induction. The system stays in the on-state as observed in panel A (error bars indicate s.d., n=3). C) EGFP fluorescence data with untagged CcrM maintenance MTase, which does not respond expression of *mf*-Lon and stays in on-state permanently in cyclic phase switching experiment conducted as described in Supplementary Fig. 19B (error bars indicate s.d., n=3).

Supplementary Tables

Statistical analysis of the data shown in Figs. 1C, 1D, 2B, 2C, 3B and 3C. The indicated p-values refer to the probability of an increase in the fluorescence or DNA methylation signals calculated using a one-sided Ttest assuming equal variance. P-values smaller than 0.05 are shaded in grey. n.a. not applicable (because the experimental signal was decreasing).

Supplementary Table 1. Statistical analysis of the data shown in Figure 1.

**Fig. 1C**	WT operon - Comparison BI with					
	AI	4 h	8 h	24 h	48 h	72 h
N	3	3	3	3	3	3
p-value	1.98E-05	8.09E-06	2.72E-05	2.83E-05	5.69E-05	5.95E-06
**Fig. 1C**	active site mutant operon - Comparison BI with					
	AI	4 h	8 h	24 h	48 h	72 h
N	3	3	3	3	3	3
p-value	1.76E-05	5.60E-04	6.33E-02	4.31E-02	4.24E-01	4.66E-01

**Fig. 1D**	WT operon - Comparison BI with					
	AI				48 h	
N	3				3	
p-value	3.27E-07				5.94E-06	
**Fig. 1D**	active site mutant operon - Comparison BI with					
	AI				48 h	
N	3				3	
p-value	0.17				0.46	

Supplementary Table 2. Statistical analysis of the data shown in Figure 2.

**Fig. 2B**	WT operon - Comparison BI with				
	AI	24 h	48 h	72 h	96 h
N	3	3	3	3	3
p-value	5.50E-03	n.a.	n.a.	n.a.	n.a.
**Fig. 2B**	active site mutant operon - Comparison BI with				
	AI	24 h	48 h	72 h	96 h
N	3	3	3	3	3
p-value	2.78E-05	n.a.	n.a.	n.a.	n.a.

**Fig. 2C**	WT operon - Comparison BI with				
	AI	24 h	48 h	72 h	96 h
N	3	3	3	3	3
p-value	1.31E-05	3.53E-05	2.57E-05	2.91E-05	1.61E-04
**Fig. 2C**	active site mutant operon - Comparison BI with				
	AI	24 h	48 h	72 h	96 h
N	3	3	3	3	3
p-value	1.87E-06	n.a.	n.a.	n.a.	n.a.

Supplementary Table 3. Statistical analysis of the data shown in Figure 3.

**Fig. 3B**	WT operon - Comparison BI with			
	AI	12 h	24 h	48 h
N	2	2	2	2
p-value	1.87E-05	1.93E-03	3.58E-02	3.83E-03
**Fig. 3B**	active site mutant operon - Comparison BI with			
	AI	12 h	24 h	48 h
N	2	2	2	2
p-value	5.18E-03	2.02E-01	1.03E-03	2.03E-02

**Fig. 3C**	WT operon - Comparison BI with			
	AI	12 h	24 h	48 h
N	2	2	2	2
p-value	4.26E-03	7.48E-03	5.30E-03	5.36E-05
**Fig. 3C**	active site mutant operon - Comparison BI with			
	AI	12 h	24 h	48 h
N	2	2	2	2
p-value	1.50E-02	6.20E-02	1.80E-04	1.72E-02

Supplementary Note

Supplementary Note 1: Memory system I annotated DNA sequence.

```
LOCUS       Exported                6393 bp ds-DNA     circular SYN 13-JAN-2017
DEFINITION  synthetic circular DNA
ACCESSION   .
VERSION     .
KEYWORDS    Memory system I GenBank
SOURCE      synthetic DNA construct
  ORGANISM  synthetic DNA construct
REFERENCE   1  (bases 1 to 6393)
  AUTHORS   Johannes
  TITLE     Direct Submission
FEATURES             Location/Qualifiers
     source          1..6393
                     /organism="synthetic DNA construct"
                     /mol_type="other DNA"
     terminator      127..206
     misc_feature    207..218
                     /note="stop codons"
     CDS             complement(219..1319)
                     /codon_start=1
                     /note="CcrM Caulobacter crescentus"
                     /translation="MGHHHHHHMKFGPETIIHGDCIEQMNALPEKSVDLIFADPPYNLQ
                     LGGDLLRPDNSKVDAVDDHWDQFESFAAYDKFTREWLKAARRVLKDDGAIWVIGSYHNI
                     FRVGVAVQDLGFWILNDIVWRKSNPMPNFKGTRFANAHETLIWASKSQNAKRYTFNYDA
                     LKMANDEVQMRSDWTIPLCTGEERIKGADGQKAHPTQKPEALLYRVILSTTKPGDVILD
                     PFFGVGTTGAAAKRLGRKFIGIEREAEYLEHAKARIAKVVPIAPEDLDVMGSKRAEPRV
                     PFGTIVEAGLLSPGDTLYCSKGTHVAKVRPDGSITVGDLSGSIHKIGALVQSAPACNGW
                     TYWHFKTDAGLAPIDVLRAQVRAGMN"
     CDS             complement(1296..1313)
                     /codon_start=1
                     /product="6xHis affinity tag"
                     /note="6xHis"
                     /translation="HHHHHH"
     misc_feature    1326..1337
                     /note="RBS"
     misc_feature    1346..1356
                     /note="ZFBS"
     misc_feature    1362..1372
                     /note="ZFBS"
     CDS             complement(1384..2103)
                     /codon_start=1
                     /note="GFP codon optimized"
                     /translation="MRKGEELFTGVVPILVELDGDVNGHKFSVSGEGEGDATYGKLTLK
                     FICTTGKLPVPWPTLVTTFGYGVQCFARYPDHMKQHDFFKSAMPEGYVQERTIFFKDDG
                     NYKTRAEVKFEGDTLVNRIELKGIDFKEDGNILGHKLEYNYNSHNVYIMADKQKNGIKV
                     NFKIRHNIEDGSVQLADHYQQNTPIGDGPVLLPDNHYLSTQSALSKDPNEKRDHMVLLE
                     FVTAAGITHGMDELYK*"
     misc_feature    2111..2123
                     /note="rbs"
     misc_feature    2140..2145
                     /note="-10"
     misc_feature    2153..2163
                     /note="ZFBS"
     misc_feature    2163..2168
                     /note="-35"
     misc_feature    2169..2179
                     /note="ZFBS"
     misc_feature    2253..2263
                     /note="ZFBS"
     misc_feature    2269..2279
                     /note="ZFBS"
     misc_feature    2494..2502
                     /note="ZFBS"
     misc_feature    2509..2517
                     /note="ZFBS"
     CDS             2568..2570
                     /codon_start=1
                     /gene="lacI"
                     /product="lac repressor"
```

```
                     /note="M"
                     /note="The lac repressor binds to the lac operator to
                     inhibit transcription in E. coli. This inhibition can be
                     relieved by adding lactose or
                     isopropyl-beta-D-thiogalactopyranoside (IPTG)."
                     /translation="M"
     CDS             2571..2594
                     /codon_start=1
                     /product="FLAG epitope tag, followed by an enterokinase
                     cleavage site"
                     /note="FLAG"
                     /translation="DYKDDDDK"
     CDS             2595..2852
                     /codon_start=1
                     /note="ZnF 1012 (seq)"
                     /translation="SRPHERPFQCRICMRNFSQASNLTRHTRTHTGEKPFQCRICMRNF
                     SQQTNLTRHLRTHTGEKPFQCRICMRNFSQTTHLSRHLRTH"
     CDS             2853..2987
                     /codon_start=1
                     /note="coiled coil"
                     /translation="LRGSSAQSGPGRMKQIEDKLEEILSKLYHIENELARIKKLLGER"
     rep_origin      complement(4417..5005)
                     /direction=LEFT
                     /note="ori"
                     /note="high-copy-number ColE1/pMB1/pBR322/pUC origin of
                     replication"
     CDS             5127..5942
                     /codon_start=1
                     /gene="aph(3')-Ia"
                     /product="aminoglycoside phosphotransferase"
                     /note="KanR"
                     /note="confers resistance to kanamycin"
                     /translation="MSHIQRETSCSRPRLNSNMDADLYGYKWARDNVGQSGATIYRLYG
                     KPDAPELFLKHGKGSVANDVTDEMVRLNWLTEFMPLPTIKHFIRTPDDAWLLTTAIPGK
                     TAFQVLEEYPDSGENIVDALAVFLRRLHSIPVCNCPFNSDRVFRLAQAQSRMNNGLVDA
                     SDFDDERNGWPVEQVWKEMHKLLPFSPDSVVTHGDFSLDNLIFDEGKLIGCIDVGRVGI
                     ADRYQDLAILWNCLGEFSPSLQKRLFQKYGIDNPDMNKLQFHLMLDEFF"
     rep_origin      complement(6035..97)
                     /direction=LEFT
                     /note="f1 ori"
                     /note="f1 bacteriophage origin of replication; arrow
                     indicates direction of (+) strand synthesis"
ORIGIN
        1 gcgaaaggag cgggcgctag ggcgctggca agtgtagcgg tcacgctgcg cgtaaccacc
       61 acacccgccg cgcttaatgc gccgctacag ggcgcgtccc attcgccaat ccggatatag
      121 ttcctcgaga gcgttcaccg acaaacaaca gataaaacga aaggcccagt ctttcgactg
      181 agcctttcgt tttatttgat gcctgggtca gtcagtcatc agttcatccc cgcccgcacc
      241 tgcgctcgca gcacatcaat cggcgccaga cccgcgtcgg tcttgaagtg ccagtaggtc
      301 cagccgttgc aggccggcgc gctctgaacg agagccccga tcttgtggat cgagcccgag
      361 aggtcgccga ccgtgatcga gccgtccgga cggaccttgg ccacgtgcgt gcccttgctg
      421 cagtaaagcg tgtcgcccgg cgacagtagg ccggcctcga cgatggtgcc gaacggcacg
      481 cgcggctcgg cgcgcttgga gcccatgacg tccaggtctt cgggcgcgat cggcacgacc
      541 ttggcgatgc gggccttggc gtgctcgagg tattcggcct cgcgctcgat gccgatgaac
      601 ttacggccta ggcgcttggc ggccgcgccg gtggtgccga cgccgaagaa cggatccagg
      661 atcacgtcgc ccggcttcgt cgtcgacagg atgacgcggt agagcagggc ctcgggcttc
      721 tgggtcgggt gggctttctg gccgtcggcg cccttgatgc gctcctcgcc ggtgcacagc
      781 gggatggtcc agtccgagcg catctgcacc tcgtcattgg ccatcttcag ggcgtcgtag
      841 ttgaaggtgt agcgcttggc gttctggctc ttggaggccc agatcagggt ctcgtgggcg
      901 ttggcgaagc gggtgccctt gaagttgggc atcgggttgg acttgcgcca gacgatgtcg
      961 ttgaggatcc agaagcccag gtcctgcacc gccacgccga cgcggaagat gttgtgatag
     1021 ctgccgatca cccagatcgc gccgtcgtcc ttcagaacgc gacgggcggc cttcagccac
     1081 tcgcgggtga acttgtcgta ggcggcgaag ctttcgaact ggtcccagtg gtcgtcgacc
     1141 gcgtcgacct tggaattgtc gggacgcagg aggtccccgc ccagctgcag attatagggc
     1201 ggatcggcga agatcaggtc gaccgacttc tcgggcaggg cgttcatctg ctcgatgcag
     1261 tcgccgtgga tgatggtttc cggcccgaac ttcatatgat gatgatgatg atggcccatc
     1321 tagtatttcc cctctttctc tagacgattc ttctcctcat tggagaagaa tcatttctag
     1381 aggttatcac ttgtacagtt cgtccatacc atgcgtaatg cccgctgcgg tgacgaattc
     1441 cagcagaacc atgtgatcac gtttttcgtt gggatctttc gacagagcac tttgtgtgct
     1501 caagtaatga ttatccggca gcaacactgg accatcaccg atcggggtgt tttgctgata
     1561 atggtcagcc agctgcacac tgccgtcttc gatgttgtgg cgaattttga aattgacctt
     1621 gataccgttt ttctgtttat cagccatgat gtacacatta tggctattat agttgtactc
     1681 aagtttatga cccagaatgt tgccatcttc tttaaaatcg ataccttta attcgatgcg
     1741 gttcaccaga gtatcgcctt cgaatttaac ttcggcacgg gtcttgtagt taccatcgtc
```

1801 tttaaaaaag atggtgcgct cttgcacata accttccggc atagcacttt tgaaaaaatc
1861 gtgttgtttc atatgatcgg ggtagcgagc aaaacactga acgccgtaac cgaaggtcgt
1921 taccagagtt ggccaaggta caggaagttt gcccgtcgta cagataaatt tcagagtaag
1981 cttgccgtag gtagcgtcac cttcgccctc accgctaacg gagaatttgt ggccgttaac
2041 atcaccatcc agctcgacca ggatggggac gacacccgta aacagctcct cccctttgcg
2101 catggtatat ctttcctgtg tgacgtgaac ttggctagca taatacctag ggattcttc
2161 tccgtaaagg agaagaatct cgagatctcg atcctctacg ccggacgcat cgtggccggc
2221 atcaccggcg ccacaggtgc ggttgctggc gcgattcttc tccaggtcgg agaagaatct
2281 caccgatggg gaagatcggg ctcgccactt cgggctcatg agcgcttgtt tcggcgtggg
2341 tatggtggca ggccccgtgg ccgggggact gttgggcgcc atctccttgc atgcaccatt
2401 ccttgcggcg gcggtgctca acggcctcaa cctactactg ggctgcttcc taatgcagga
2461 gtcgcataag ggagagcgtc gagatagcgg catttcttct ccttgacagg agaagaagcg
2521 gtatggcatg atagcgcccg gaagagagtc aattcagggt ggtgaatgtg gactacaaag
2581 acgatgatga caaatctaga ccacacgaac gccctttca gtgccgtatc tgcatgcgta
2641 acttttcaca ggcgagcaac ctgacgcgtc atacgcgcac ccataccggc gaaaagccgt
2701 tccagtgtcg catttgtatg cgcaactttt cacaacaaac caacctgaca cgccatttgc
2761 gtacccacac gggtgagaaa ccgttccaat gccgtatttg catgcgcaac ttctcgcaaa
2821 ccacacacct gagtcgtcac cttcgcacgc atctgcgtgg atcctctgca cagtccggtc
2881 cgggacgtat gaaacaaatc gaagacaaac tggaagagat tttgtctaaa ctgtaccata
2941 ttgaaaatga attggcgcgc atcaagaaac tgctcggtga acgctgagcg caacgcaatt
3001 aatgtaagtt agctcactca ttaggcaccg ggatctcgac cgatgccctt gagagccttc
3061 aacccagtca gctccttccg gtgggcgcgg ggcatgacta tcgtcgccgc acttatgact
3121 gtcttcttta tcatgcaact cgtaggacag gtgccggcag cgctctgggt cattttcggc
3181 gaggaccgct ttcgctggag cgcgacgatg atcggcctgt cgcttgcggt attcggaatc
3241 ttgcacgccc tcgctcaagc cttcgtcact ggtcccgcca ccaaacgttt cggcgagaag
3301 caggccatta tcgccggcat ggcggcccca cgggtgcgca tgatcgtgct cctgtcgttg
3361 aggacccggc taggctggcg gggttgcctt actggttagc agaatgaatc accgatacgc
3421 gagcgaacgt gaagcgactg ctgctgcaaa acgtctgcga cctgagcaac aacatgaatg
3481 gtcttcggtt tccgtgtttc gtaaagtctg gaaacgcgga agtcagcgcc ctgcaccatt
3541 atgttccgga tctgcatcgc aggatgctgc tggctaccct gtggaacacc tacatctgta
3601 ttaacgaagc gctggcattg accctgagtg atttttctct ggtcccgccg catccatacc
3661 gccagttgtt taccctcaca acgttccagt aaccgggcat gttcatcatc agtaacccgt
3721 atcgtgagca tcctctctcg tttcatcggt atcattaccc ccatgaacag aaatccccct
3781 tacacggagg catcagtgac caaacaggaa aaaaccgccc ttaacatggc ccgctttatc
3841 agaagccaga cattaacgct tctggagaaa ctcaacgagc tggacgcgga tgaacaggca
3901 gacatctgtg aatcgcttca cgaccacgct gatgagcttt accgcagctg cctcgcgcgt
3961 ttcggtgatg acggtgaaaa cctctgacac atgcagctcc cggagacggt cacagcttgt
4021 ctgtaagcgg atgccgggag cagacaagcc cgtcagggcg cgtcagcggg tgttggcggg
4081 tgtcggggcg cagccatgac ccagtcacgt agcgatagcg gagtgtatac tggcttaact
4141 atgcggcatc agagcagatt gtactgagag tgcaccatat atgcggtgtg aaataccgca
4201 cagatgcgta aggagaaaat accgcatcag gcgctcttcc gcttcctcgc tcactgactc
4261 gctgcgctcg gtcgttcggc tgcggcgagc ggtatcagct cactcaaagg cggtaatacg
4321 gttatccaca gaatcagggg ataacgcagg aaagaacatg tgagcaaaag gccagcaaaa
4381 ggccaggaac cgtaaaaagg ccgcgttgct ggcgtttttc cataggctcc gcccccctga
4441 cgagcatcac aaaaatcgac gctcaagtca gaggtggcga aacccgacag gactataaag
4501 ataccaggcg tttccccctg gaagctccct cgtgcgctct cctgttccga ccctgccgct
4561 taccggatac ctgtccgcct ttctcccttc gggaagcgtg gcgctttctc atagctcacg
4621 ctgtaggtat ctcagttcgg tgtaggtcgt tcgctccaag ctgggctgtg tgcacgaacc
4681 ccccgttcag cccgaccgct gcgccttatc cggtaactat cgtcttgagt ccaacccggt
4741 aagacacgac ttatcgccac tggcagcagc cactggtaac aggattagca gagcgaggta
4801 tgtaggcggt gctacagagt tcttgaagtg gtggcctaac tacggctaca ctagaaggac
4861 agtatttggt atctgcgctc tgctgaagcc agttaccttc ggaaaaagag ttggtagctc
4921 ttgatccggc aaacaaacca ccgctggtag cggtggtttt tttgtttgca agcagcagat
4981 tacgcgcaga aaaaaaggat ctcaagaaga tcctttgatc ttttctacgg ggtctgacgc
5041 tcagtggaac gaaaactcac gttaagggat tttggtcatg aacaataaaa ctgtctgctt
5101 acataaacag taatacaagg ggtgttatga gccatattca acgggaaacg tcttgctcta
5161 ggccgcgatt aaattccaac atggatgctg atttatatgg gtataaatgg gctcgcgata
5221 atgtcgggca atcaggtgcg acaatctatc gattgtatgg gaagcccgat gcgccagagt
5281 tgtttctgaa acatggcaaa ggtagcgttg ccaatgatgt tacagatgag atggtcagac
5341 taaactggct gacggaattt atgcctcttc cgaccatcaa gcattttatc cgtactcctg
5401 atgatgcatg gttactcacc actgcgatcc ccgggaaaac agcattccag gtattagaag
5461 aatatcctga ttcaggtgaa aatattgttg atgcgctggc agtgttcctg cgccggttgc
5521 attcgattcc tgtttgtaat tgtcctttta acagcgatcg cgtatttcgt ctcgctcagg
5581 cgcaatcacg aatgaataac ggtttggttg atgcgagtga ttttgatgac gagcgtaatg
5641 gctggcctgt tgaacaagtc tggaaagaaa tgcataaact tttgccattc tcaccggatt
5701 cagtcgtcac tcatggtgat ttctcacttg ataaccttat ttttgacgag gggaaattaa
5761 taggttgtat tgatgttgga cgagtcggaa tcgcagaccg ataccaggat cttgccatcc
5821 tatggaactg cctcggtgag ttttctcctt cattacagaa acggcttttt caaaaatatg
5881 gtattgataa tcctgatatg aataaattgc agtttcattt gatgctcgat gagtttttct
5941 aagaattaat tcatgagcgg atacatattt gaatgtattt agaaaaataa acaaataggg
6001 gttccgcgca catttccccg aaaagtgcca cctgaaattg taaacgttaa tattttgtta
6061 aaattcgcgt taaatttttg ttaaatcagc tcatttttta accaataggc cgaaatcggc
6121 aaaatccctt ataaatcaaa agaatagacc gagatagggt tgagtgttgt tccagtttgg

```
6181 aacaagagtc cactattaaa gaacgtggac tccaacgtca aagggcgaaa aaccgtctat
6241 cagggcgatg gcccactacg tgaaccatca ccctaatcaa gtttttttggg gtcgaggtgc
6301 cgtaaagcac taaatcggaa ccctaaaggg agcccccgat ttagagcttg acggggaaag
6361 ccggcgaacg tggcgagaaa ggaagggaag aaa
//
```

Supplementary References

1 Rathert, P. *et al.* Reversible Inactivation of the CG Specific SssI DNA (Cytosine-C5)-Methyltransferase with a Photocleavable Protecting Group. *Chembiochem* **8**, 202-207 (2007).

2 Gama-Castro, S. *et al.* RegulonDB version 9.0: high-level integration of gene regulation, coexpression, motif clustering and beyond. *Nucleic acids research* **44**, D133-D143 (2016).

3 Seeman, N. C., Rosenberg, J. M. & Rich, A. Sequence-specific recognition of double helical nucleic acids by proteins. *Proceedings of the National Academy of Sciences* **73**, 804-808 (1976).

4 Aggarwal, A. K., Rodgers, D. W., Drottar, M., Ptashne, M. & Harrison, S. C. Recognition of a DNA operator by the repressor of phage 434: a view at high resolution. *Science* **242**, 899 (1988).

5 Wolfe, S. A., Nekludova, L. & Pabo, C. O. DNA recognition by Cys2His2 zinc finger proteins. *Annual review of biophysics and biomolecular structure* **29**, 183-212 (2000).

6 Wright, D. A. *et al.* Standardized reagents and protocols for engineering zinc finger nucleases by modular assembly. *Nature protocols* **1**, 1637-1652 (2006).

7 Albu, R. F., Jurkowski, T. P. & Jeltsch, A. The Caulobacter crescentus DNA-(adenine-N6)-methyltransferase CcrM methylates DNA in a distributive manner. *Nucleic acids research* **40**, 1708-1716 (2012).

8 Maier, J. A., Albu, R. F., Jurkowski, T. P. & Jeltsch, A. Investigation of the C-terminal domain of the bacterial DNA-(adenine N6)-methyltransferase CcrM. *Biochimie* **119**, 60-67 (2015).
